# An Experimental Study on Pile Spacing Effects under Lateral Loading in Sand

**DOI:** 10.1155/2013/734292

**Published:** 2013-12-25

**Authors:** Mahdy Khari, Khairul Anuar Kassim, Azlan Adnan

**Affiliations:** Department of Geotechnics and Transportation, Faculty of Civil Engineering, Universiti Teknologi Malaysia, Skudai, 81300 Johor Bahru, Malaysia

## Abstract

Grouped and single pile behavior differs owing to the impacts of the pile-to-pile interaction. Ultimate lateral resistance and lateral subgrade modulus within a pile group are known as the key parameters in the soil-pile interaction phenomenon. In this study, a series of experimental investigation was carried out on single and group pile subjected to monotonic lateral loadings. Experimental investigations were conducted on twelve model pile groups of configurations 1 × 2, 1 × 3, 2 × 2, 3 × 3, and 3 × 2 for embedded length-to-diameter ratio *l/d* = 32 into loose and dense sand, spacing from 3 to 6 pile diameter, in parallel and series arrangement. The tests were performed in dry sand from Johor Bahru, Malaysia. To reconstruct the sand samples, the new designed apparatus, Mobile Pluviator, was adopted. The ultimate lateral load is increased 53% in increasing of *s/d* from 3 to 6 owing to effects of sand relative density. An increasing of the number of piles in-group decreases the group efficiency owing to the increasing of overlapped stress zones and active wedges. A ratio of *s/d* more than *6d* is large enough to eliminate the pile-to-pile interaction and the group effects. It may be more in the loose sand.

## 1. Introduction

Superstructures are supported by pile foundations so that it had its origin in prehistoric time. These foundations may be subjected to significant horizontal loads such as dynamic and static loadings. Two criteria shall be controlled to satisfy of functioning such structures: (1) their deflection which must be within the permissible limit and (2) safety of pile against ultimate failure. The behavior of the pile group and the single pile is usually different owing to the impacts of the pile-to-pile interaction (so called shadowing effects). In addition, soil-pile coupling behavior is important when the load transfer occurs [1]. Evaluation of the pile group behavior and the soil-pile interaction has developed by several investigators in experimental and analytical modeling [2–4]. Existing methods of the analytical modeling can be classified into numerical approaches, Beam on Nonlinear Winkler Foundation method (BNWF), and simplified formulations [5]. Although most of these approaches are attended on evaluation of the stiffness of the soil-pile system, they are less focused on the bending moment and the lateral resistance of the group.

It is worth noting that the estimations of ultimate lateral resistance and lateral subgrade modulus within a pile group are known as they are the key parameters in the soil-pile interaction phenomenon. Several theoretical methods have been developed to determine these parameters in cohesionless soils. However, the predictions of these approaches are often different. On the other hand, the laterally loaded pile group behavior has received a little attention. Moreover, the experimental data on the determination of active pile length and bending moment are inadequate. Therefore, it is necessary to increase the experimental data for the response of the pile group under lateral loads.

This paper presents the results of a series of experimental investigations carried out on single and grouped piles subjected to the monotonic lateral loads in Johor Bahru sand in the southern portion of Malaysia. Emphasis was focused on group efficiency and load-deflection behavior owing to the influence of relative density, size group, and pile spacing.

## 2. Brief Review

As mentioned in the foregoing section, the shadowing phenomenon affects the pile behavior within the group under the lateral loading [6]. Although many researchers have studied the ultimate lateral resistance and deflection of the pile group to a lateral loading, they are complex due to the interaction between the surrounding soil and the pile [7].

In 1962, Prakash carried out the pile group behavior under the lateral loading using aluminum pipes (od = 12.7 mm; *d* = pile diameter) in the medium sand. Based on these tests, it was stated that the sum of pile capacities was more than that within the group when the spacing center-to-center of piles was less than 3*d* and 8*d* in the direction perpendicular and the direction to load, respectively. Meyerhof et al. [8] conducted tests in homogeneous sand on pile groups and rigid single pile under central inclined loads. The bored piles were tested by Franke [9] in the experimental tests. The results showed that the displacement of a group was more than a single pile in the same loading when the piles spacing was less than 6*d*. Patra and Pise [10] studied the ultimate lateral resistance on six types of configurations of pile group with different embedment length-to-diameter ratios equal to 12 and 38. Their results were compared with the results of analytical methods. Based on their report, it can be stated that the isolation spacing is six times of pile diameter for *l*/*d* = 12.

Kim and his workers [11] investigated lateral load tests on aluminum single pile (driven and drilled) in dry sand. In addition, they considered the head conditions of the piles. The lateral loads of the preinstalled were less than those of the driven piles.

Zhang et al. [12] proposed the ultimate lateral resistance in cohesionless soils. They collected the experimental data done by other researchers on rigid piles and a simple method was developed by them to predict the ultimate lateral resistance (involving of side shear resistance and frontal soil resistance) to piles considering the shape factor. Another method was developed by Prakash and Kumar [13]. In this method, load-displacement relationship was predicted by means of considering soil nonlinearity using subgrade reaction. Erdal and Laman [14] purposed the behavior of short pile subjected to lateral loads in a two-layer sand deposit. The pile modeled had an embedded length-to-diameter ratio of 4 and fabricated from steel for all the tests. Based on their results, it can be stated that the lateral load capacity of short rigid piles in the dense sand was 5 times that in loose sand.

## 3. Experimental Setup

**Figure 1:**
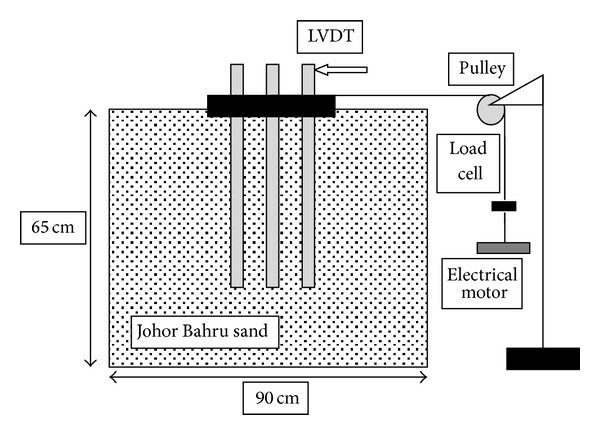
Side view of experimental setup.

The schematic diagram of the test setup is shown in Figure 1. The model tests were performed in a rectangular soil tank with dimensions of 900 mm in length, 700 mm in width, and 65 mm in height. To consider the boundary conditions, the size of the soil tank was extended up to 8–12*d* (*d* = pile diameter) and 3-4*d* in the direction and perpendicular to the lateral loading, respectively [15]. In additional, to minimize the influence of box boundaries, the soil thickness was kept below the pile tip at least 6*d*.

The model piles with an open end and hollow circular section were fabricated from aluminum alloy tubes (*E*_*p*_ = 69.8 GPa) of 15.88 mm out diameter, 1 mm wall thickness and an embedded depth of 500 mm. It is worth noting that, for the pile properties and the selected soil, pile behaves as flexible pile.

Three plates made of steel were used as pile cap for different spacing. To satisfy fixed head conditions, the piles were passed through exiting holes in the cap and then screwed to angle profiles (length = 50 mm) welded on these holes. Lateral loads were applied to the model piles using a 650 N capacity electric motor through a pulley supported by a loading platform with flexible wire attached to the cap.

The horizontal deflection of the pile group was measured by means of two Linear Variable Differential Transducers (LVDT) to the angle profiles of the two corner piles. The rotation of cap was determined from axial displacement measured by other two LVDTs fixed on front and behind of the cap in load direction. A load cell was placed between the flexible wire and electric motor to monitor the total loads applied to the pile cap.

## 4. Soil Properties and Sample Preparation

**Figure 2:**
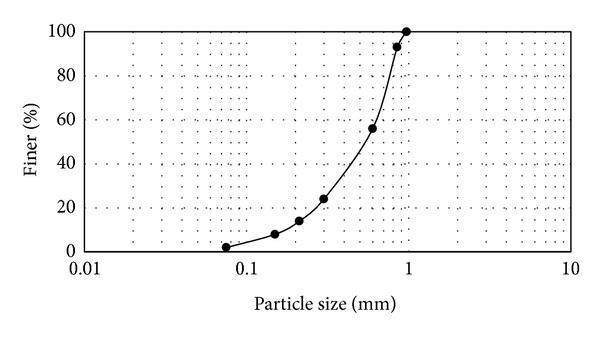
Gradation curve of the Johor Bahru sand.

The tests were conducted in dried sand (in the laboratory temperature) from Johor Bahru sand. The sampled sand was classified as SP, according to the Unified Soil Classification System (USCS). The medium diameter (*D*_50_) and uniformity coefficient (*C*_*u*_) of sand were 0.532 and 0.17 mm, respectively, and particle sizes in a range of 0.075–0.97 mm with the gradation are shown in Figure 2. Based on a standard density test, minimum and maximum unit weights of sand were 13.74 kN/m^3^ and 16.38 kN/m^3^.

To reconstruct the sand samples, several methods have been developed by investigators such as vibration, tamping, and pluviation [16]. The prepared samples using the pluviation and tamping technique often result in a specimen of homogenous and nonuniform density, respectively. Based on this defect, the newly designed Mobile Pluviator was utilized in this research to reconstruct the dry sandy soil samples using the dry pluviation method. The newly Mobile Pluviator developed was consisting mainly of a soil bin (hopper), the diffuser system (the three sieves), sand collector, and a fixing device to set up these components so as the whole of the system was carried by a moveable steel frame. The interchangeable circular wood plates (shutter plates) were installed in the bottom of the sand hopper. The four patterns of the shutter plates were formed in a manner of the distribution differently of the holes for the sake of control of the rate of the soil discharge. While the apparatus was movable, the different factors were examined to obtain a wide range of the relative density. The falling height and the rate of pouring had the opposite effects on the relative density. Based on the results obtained, the two patterns selected consisted of 11 holes (diameter = 18 mm) and 16 holes (diameter = 10 mm) distributed evenly in the shutter to achieve the dense and the loose sand samples with relative density of 75% and 30%, respectively. The falling height was kept constant a 700 mm from the surface of the model ground which was more than the critical height so that to obtain terminal velocity. The raining was stopped when the sand rained in the soil tank was 30 mm thicker than required and then the extra soils were removed.

## 5. Test Procedure

**Figure 3:**
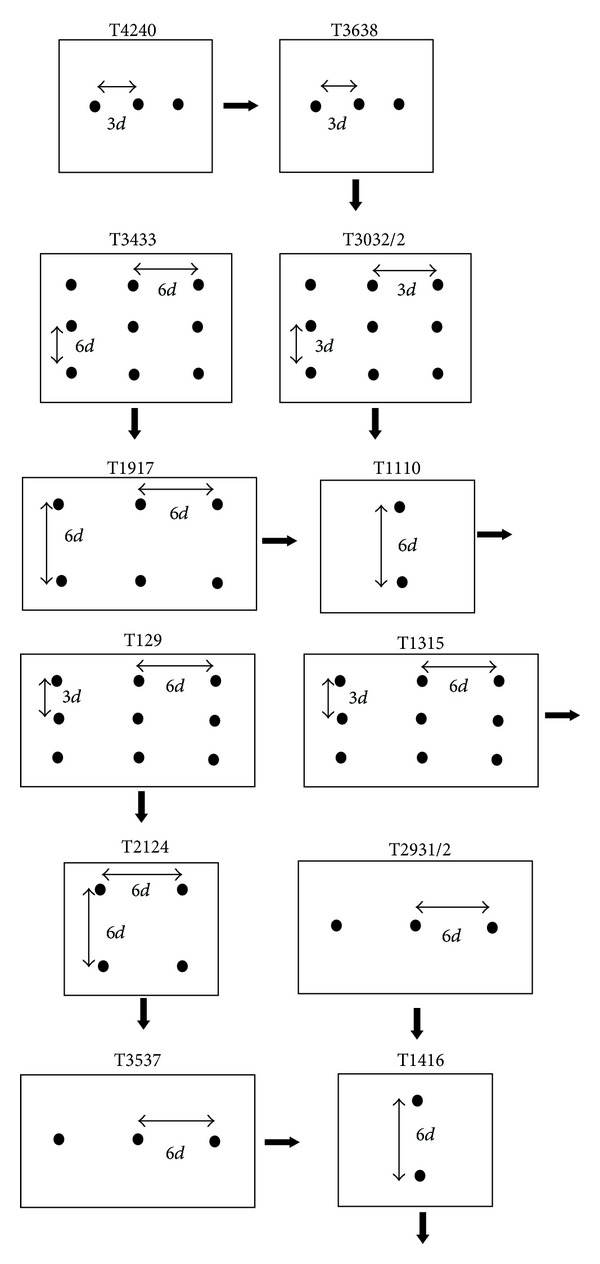
Pile group configurations and pile spacing ratio (↑ is the lateral loading direction).

Different configurations of pile groups in different spacing are shown in Figure 3. The center-to-center spacings of the piles were 6*d* and 3*d*, and embedment ratio of 32 was tested. Spacing ratio (SR = *S*_2_/*S*_1_, where *S*_2_ and *S*_1_ are the piles spacing in perpendicular and direction of lateral load applied, resp.) was equal to 0.5, 1 and 2. In addition, several tests were conducted on single pile. The piles (fixed with the cap) were first located in the center of soil tank and then were kept in a vertical statue using a supporting frame. After placing the model pile, the Mobile Pluviator apparatus was installed over soil box. To monitor uniformity and the relative density during the samples preparation, three small boxes cylinder shaped of 455 cm^3^ were placed on the surface of sample prior to sand spreading. The surface of the model ground was leveled when the required height was achieved. At least 24 hours elapsed before applying any test on the pile group. The data measured from LVDTs and load cell were stored on a computer data acquisition system.

## 6. Experimental Results and Discussion

A series of 45 tests were performed on piles to investigate the influences of soil density and different pile configurations on the ultimate lateral resistance and pile group efficiency. The pile groups were loaded in an incremental manner. The nonlinear load versus lateral displacement and vertical settlement of the pile cap could be adequately defined.

**Figure 4:**
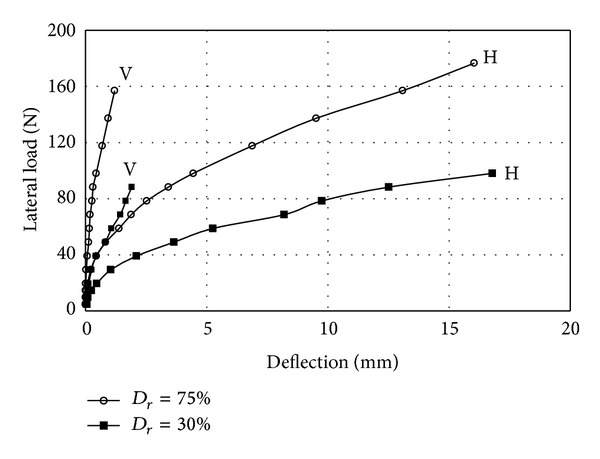
Lateral load versus deflection diagram for single pile (H = horizontal; V = vertical).

The soil density effects on single pile against the average pile deflection are presented in Figure 4. From the figure it is seen that the load-deflection curves were nonlinear and a similar trend was observed in loose and dense conditions. Vertical displacements were negligible compared to horizontal deflections and it is in agreement with previous studies which stated that soil-pile interaction could be determined separately under lateral and vertical loads. The differences of the lateral deflection increased when the relative density increased from 30% to 75% under the same moment of load. Therefore, a higher relative density will provide a stiffer resistance for pile subjected to lateral loading. This is owing to the increasing of shear strength of sand as it becomes denser. In other words, pile behavior subjected to lateral loads depends on the interaction between the surrounding soil and pile material.

**Figure 5:**
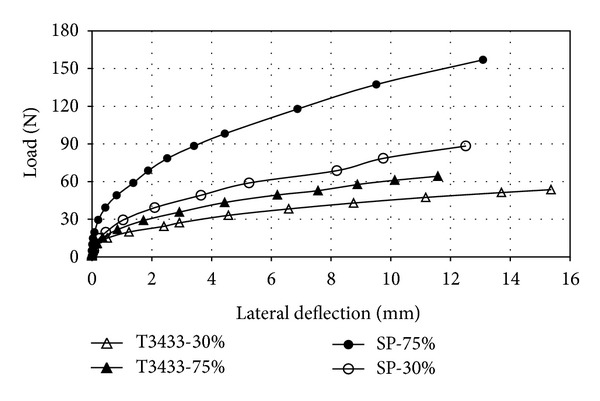
Lateral load versus lateral deflection (3 × 3 pile group; *s*/*d* = 6).

**Figure 6:**
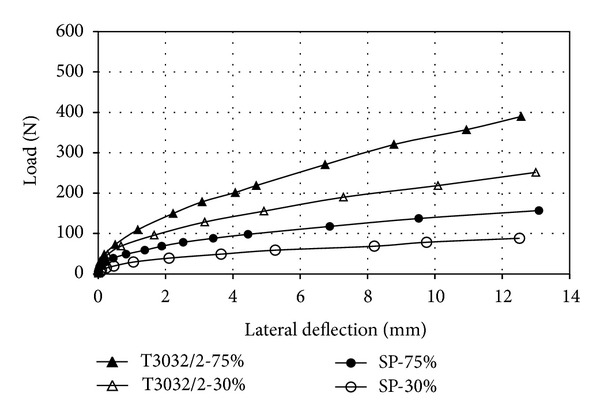
Lateral load versus lateral deflection (3 × 3 pile group; *s*/*d* = 3).

The influence of the piles' spacing on the lateral deflection and group behavior for the 3 × 3 pile group with a square arrangement are shown in Figures 5 and 6. For a particular value of lateral movement, the magnitude of lateral load decreased when the piles' spacing decreased in dense and loose sand. At the deflection of 0.1*d*, the lateral load of the pile group was about 2.90 times higher than that of the single pile in the case of 6-diameter, 1.85 times higher for *s*/*d* = 3.

**Figure 7:**
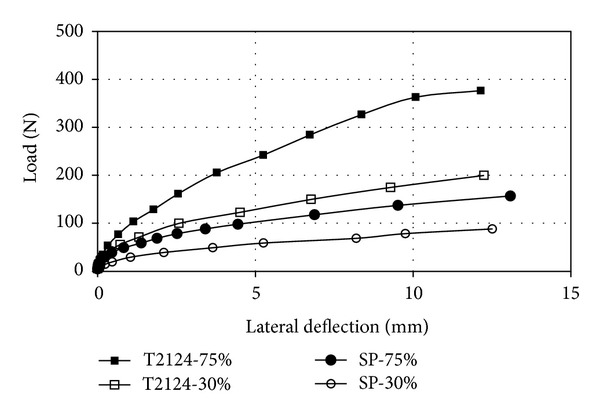
Lateral load versus lateral deflection (2 × 2 pile group; *s*/*d* = 6).

Figure 7 illustrates the influences of piles number in-group on the value of the deflection against the lateral load. Compared to Figure 5, it is observed that when the piles' spacing was the same, the magnitude of lateral load however was higher for larger groups. comparing between Figures 6 and 7, the load-deflection curves were almost similar. This may be due to the area of ground pressure in front of the pile group. This indicates that, although the number of piles contributes to the value of lateral resistance, the piles' spacing is the most significant factor.

**Figure 8:**
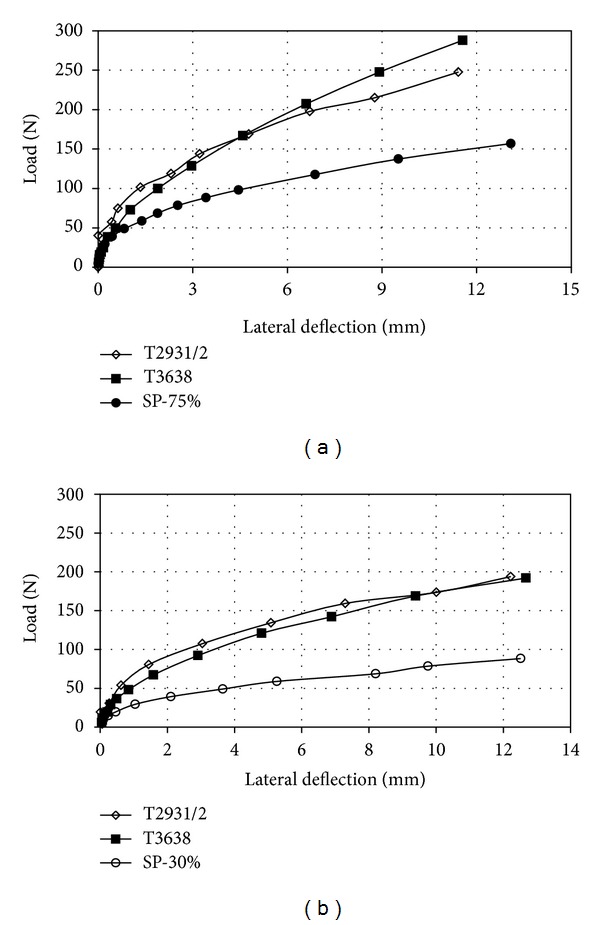
Lateral load versus lateral deflection for three-pile group in series layout; (a) *D*_*r*_ = 75% and (b) *D*_*r*_ = 30%.

**Figure 9:**
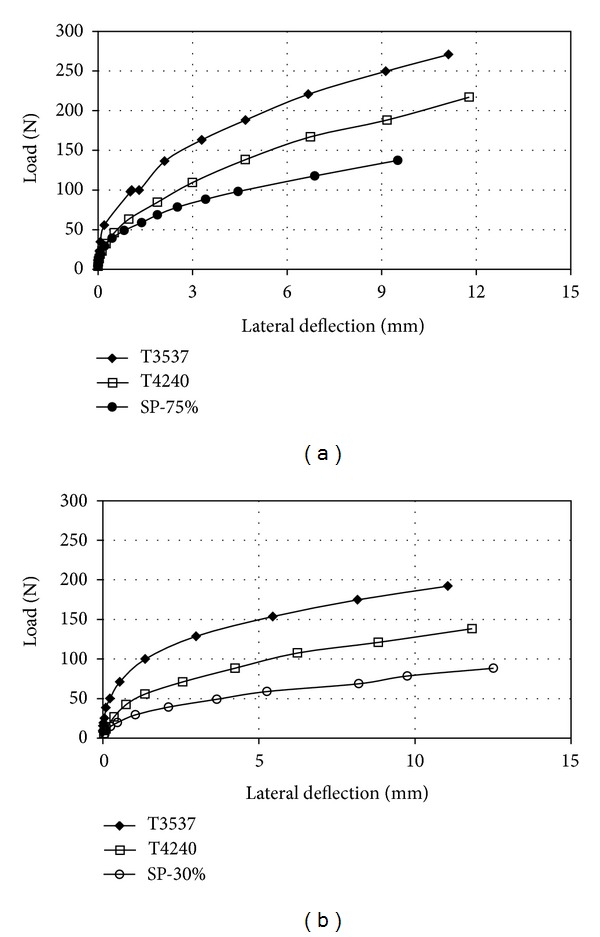
Lateral load versus lateral deflection for three-pile group in Parallel layout; (a) *D*_*r*_ = 75% and (b) *D*_*r*_ = 30%.

Figures 8 and 9 illustrate the behavior of the load-deflection of pile group in both series and parallel arrangements were investigated for a three-piles group in the spacing of the center-to-center piles of 3*d* and 6*d*. From these Figures, it can be seen that the piles' deflection in parallel arrangement was less than that in series arrangement under a given lateral load. The higher lateral load capacities in parallel arrangement was governed by the increased passive pressure zone existed in front of the pile group. A similar comparison was made for different relative densities of soil, which shows that a similar phenomenon occurred.

It should be noted as shown in Figures 8 and 9 that the effect of the stressed zone around piles for series arrayed piles was less than that for parallel arrangement. However, both stress zones may be dependent on the dimensions and the elastic modulus of the piles. Since the piles were assumed flexible, the failure of the surrounding soil will be earlier as compared to the piles.

## 7. Ultimate Lateral Resistance

**Figure 10:**
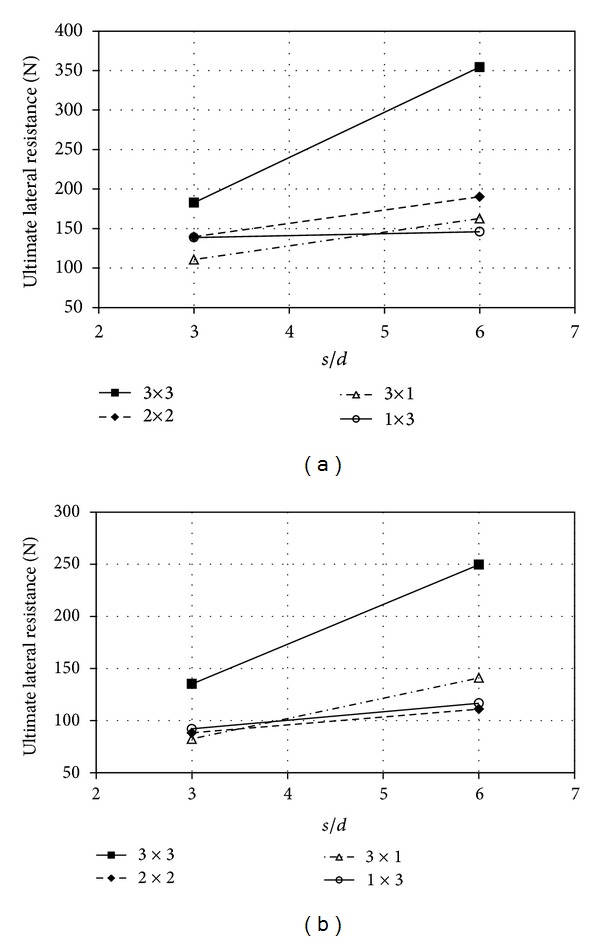
Ultimate lateral load versus pile spacing; (a) *D*_*r*_ = 75% and (b) *D*_*r*_ = 30%.

The ultimate lateral resistance in the different arrangements of pile groups was estimated by the load-deflection curves. The soil resistance to piles under lateral load may be involving of the side friction and the frontal normal reaction [17]. However, these two reactions are dependent on shape factor taking in account nonuniform distribution of earth pressure in front of pile and lateral shear drag. There are several methods to estimate the ultimate lateral resistance such as double tangent and log-log method. In this study, the ultimate lateral resistance was taken as the load corresponding to the reference deflection of 0.2*d* on the load-deflection curves [18]. The results obtained exhibit that the increasing rate of deflection was reached at about 0.2–0.35*d*.  Figure 10 shows the influence of the piles' spacing versus ultimate lateral resistance. The ultimate lateral load was constant with an increase from 3*d* to 6*d* in parallel arrangement of piles for group 1 × 3 in dense sand. However, the increasing can be observed more than that in series arrangement of piles. From the figure, It is worth noting that the relative density affects ultimate resistance because of passive pressure zone existed in front of the pile group. The piles' spacing in the perpendicular direction to load applied may affect the ultimate resistance load due to the stressed zone in front of the pile group. The ultimate lateral resistance of single piles was 84.013 and 44.5 (N) for loose and dense sand, respectively. With note to the ultimate load in group and single pile, the effects of the shadowing phenomenon can be observed so as the increasing of the pile spacing causes the same in group and individual. The ultimate lateral load in single pile was about 25% of the ultimate load for 3 × 3 pile group (T3433) while this percentage for T3032/3 was about 47%. In fact, with increasing of the pile spacing from 3*d* to 6*d*, the value of ultimate lateral load about 0.53% is increased.

## 8. Group Efficiency

Variation of the pile group resistance at a given deflection is expressed by group efficiency (*η*) and is calculated as follows:

(1) $$\begin{array}{c} \eta =\frac{Q_{\text{LG}}}{n_{1}n_{2}Q_{\text{LS}}}, \end{array}$$

where *Q*_LG_ and *Q*_LS_ are ultimate lateral capacity of pile group and single pile, respectively. *n*_1_  is  number of rows in a pile group; *n*_2_ is number of columns in a pile group.

Wakai et al. [19] performed the laboratory tests on a 3 × 3 pile group with free and fixed head conditions (*s* = 2.5*d*). The group efficiency was estimated 0.45–0.70 at the deflection of 0.1*d*. However, the group efficiency obtained based on the ultimate lateral loading can be higher than that at a given deflection. Kim and Yoon [20] carried out the static loading tests on the different pile arrangements. They calculated the group efficiency when the deflection was reached 0.1*d*. In 3 × 3 pile group, the coefficient was 0.4–0.7 and 0.5–1.04 for the medium dense and the medium sand, respectively.

Gandhi and Selvam [21] stated that, at the 10 mm displacement, pile behavior is crossed through elastic to plastic range. They considered this deflection to estimate the group efficiency. Based on their results, the efficiency increases with an increase in the *s*/*d* ratio and this raising can be due to the increasing of the overlapping zones.

**Figure 11:**
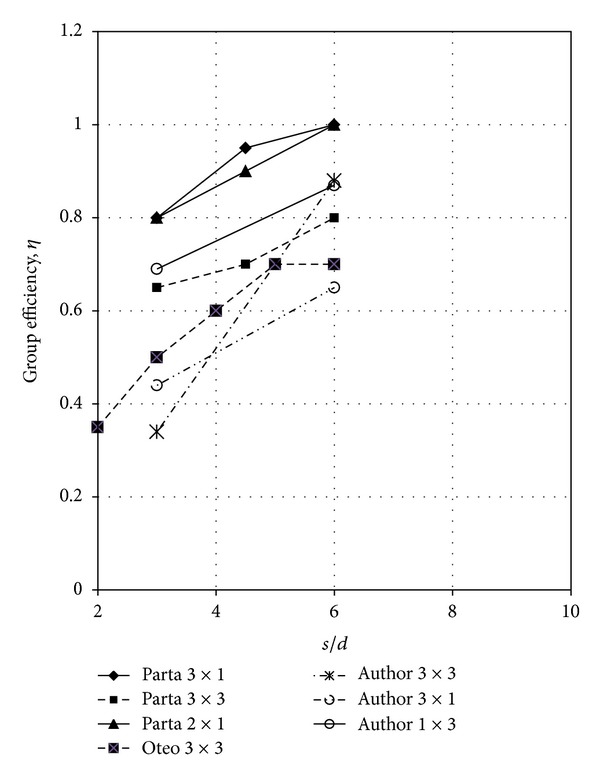
Comparison of group efficiencies.

Patra and Pise [10] and Oteo [22] carried out a series of tests on different configurations of pile groups under the lateral loading. Oteo reported model tests on 3 × 3 piles group in medium send. Patra and Pise reported the groups' efficiencies for 2 × 1, 3 × 1, 2 × 2, and 3 × 2 for pile spacing from 3*d* to 6*d*. As Figure 11 shows, the experimental results in this study for 3 × 1 pile group at 3*d* and 6*d* were about 50% less than those the reported by Patra and Pise. However, the measured group efficiencies were in good agreement with those of Oteo.

**Figure 12:**
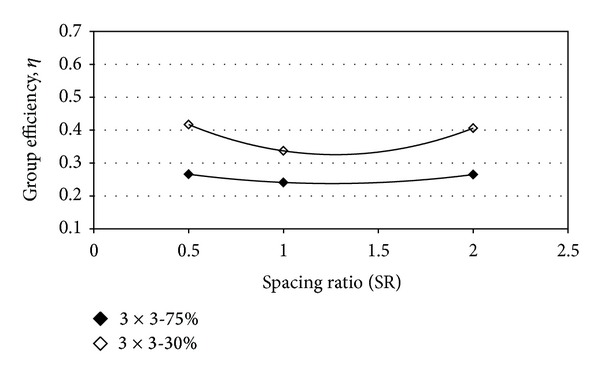
Group efficiency versus spacing ratio of piles; (a) *D*_*r*_ = 75% and (b) *D*_*r*_ = 30%.

**Figure 13:**
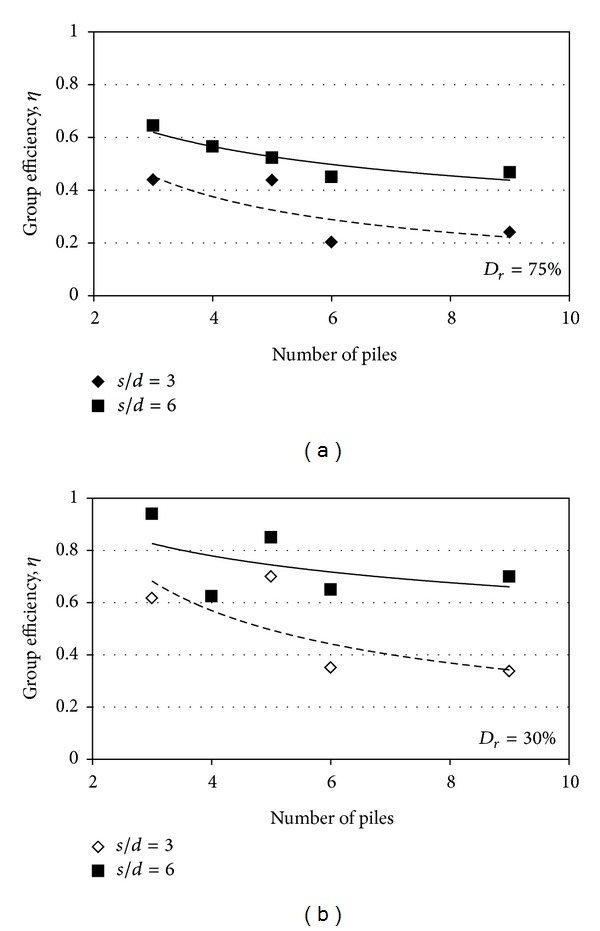
Group efficiency VS number of piles; (a)  *D*_*r*_ = 75% and (b) *D*_*r*_ = 30%.

The variation group efficiency against of spacing ratio (SR = *S*_2_/*S*_1_, where *S*_2_ and *S*_1_ are the piles spacing in perpendicular and direction of lateral load applied, resp.) is presented in Figure 12 for 3 × 3 piles group in the different relative densities. It can be stated that the group efficiency was decreased about 0.35 and 0.25 in the loose and the dense sand where *S*_1_ = *S*_2_. However, this value was increased for *S*_2_ ≠ *S*_1_. The group efficiencies were the same for the *S*_2_/*S*_1_ ratio almost equal to 0.5 and 2. The group efficiency was higher in relative density by 30%. For a 3 × 3 piles group, the observed efficiency was about 0.23–0.28% and 0.32–.41% for *D*_*r*_ = 75% and *D*_*r*_ = 30%, respectively. As Figure 13 shows, the group efficiency was decreased with an increase in the number of piles arrayed in group. This decreasing with an increase of the number of piles in pile spacing of 6*d* and 3*d* was almost the same. However, the group efficiency was about 0.68–0.84% for *s* = 6*d* and 0.35–0.68% for *s* = 3*d*.

Pise and Patra [10] carried out a series of the tests for 3 × 3, 3 × 1, 2 × 2, and 2 × 1 piles groups. The efficiencies obtained were 0.752–1.0 and 0.9–1.2 for the 3 × 2 and the 3 × 3-piles group, respectively. These efficiencies were higher (about 42%–78%) than those obtained in this study for *s*/*d* = 3 and 6.

## 9. Conclusions

The behavior of single pile and grouped is believed to be understood, especially for soils where the subgrade modulus is independent of time. Based on this demand, a series of tests were carried out on pile group under lateral static loading in sandy soils. A new method of the reconstruction of sand samples was developed for large area of samples. Based on the results of present experiment, the following conclusions are drawn.

1. Load-deflection curves were estimated with scaling factors to determine the ultimate lateral resistance of group. The qualitative and quantitative effects of the relative density of the sand have been carried out. The ultimate lateral load was increased 53% in increasing of *s*/*d* from 3 to 6.
2. The subgrade modulus decreased with increasing deflection. Width and pile stiffness were two important factors effective on this decreasing.
3. Vertical deflection of pile group can be neglected with comparison to horizontal deflection under the lateral loading.
4. The increase of the number of piles in-group decreased group efficiency owing to the increased overlapping zones and active wedges.
5. A ratio of *s*/*d* more than 6*d* was large enough to eliminate the pile-to-pile interaction and the group effects. It may be more in the loose sand.
6. Flexible piles of series arrayed were more resistant than those parallel arrayed to lateral loadings.
